# Venous Congestive Myelopathy and Secondary Infarction: A Delayed Diagnosis of Spinal Dural Arteriovenous Fistula

**DOI:** 10.7759/cureus.89076

**Published:** 2025-07-30

**Authors:** Ahmad Elbaghdady, Shafaq Ismail, Jerry Brown Njoh Aseneh, Sadaf Chara, Sapna Philip

**Affiliations:** 1 Acute Medicine, King's Mill Hospital, Sherwood Forest Hospitals NHS Foundation Trust, Sutton-in-Ashfield, GBR; 2 Critical Care, King's Mill Hospital, Sherwood Forest Hospitals NHS Foundation Trust, Sutton-in-Ashfield, GBR; 3 Pulmonology, King's Mill Hospital, Sherwood Forest Hospitals NHS Foundation Trust, Sutton-in-Ashfield, GBR; 4 General Internal Medicine, King's Mill Hospital, Sherwood Forest Hospitals NHS Foundation Trust, Sutton-in-Ashfield, GBR

**Keywords:** owl's sign, spinal dural arteriovenous fistula, spinal infarction, transverse myelitis, venous congestive myelopathy

## Abstract

A man in his 70s with known vascular risk factors presented with acute onset of bilateral lower limb weakness and urinary retention. Initial spinal magnetic resonance imaging (MRI) revealed a longitudinally extensive myelopathy. It was initially interpreted as transverse myelitis, prompting treatment with high-dose corticosteroids. However, the clinical response was minimal. Over the following months, progressive symptom worsening led to a repeat MRI, which showed interval progression of spinal cord edema and T2 hyperintensity from T6 to T12, which was indicative of chronic venous congestion without distinct flow voids. Subsequent spinal angiography confirmed a spinal dural arteriovenous fistula (SDAVF) at the T10-T11 level. The clinical course and imaging findings led to a diagnosis of venous congestive myelopathy (VCM), complicated by secondary spinal cord infarction (SCI) attributable to the SDAVF. After surgical disconnection of the fistula, the patient began intensive rehabilitation. Despite successful fistula closure, substantial residual neurological deficits persisted, likely due to irreversible damage from both chronic venous hypertension and secondary infarction, compounded by delayed diagnosis. This case underscores the importance of considering SDAVF in patients with progressive or steroid-refractory myelopathy. It also illustrates that VCM can lead to secondary infarction, complicating both diagnosis and prognosis.

## Introduction

Spinal dural arteriovenous fistula (SDAVF) is the most common type of spinal vascular malformation, accounting for 70%-80% of such lesions, though it remains relatively uncommon overall [1]. These fistulas primarily affect middle-aged and older men, with a predilection for the thoracolumbar spine [2]. The pathophysiology involves an acquired abnormal communication between a meningeal artery and a radicular vein within the dural nerve root sleeve [1]. This shunt allows retrograde venous drainage under arterial pressure into the perimedullary venous plexus of the spinal cord. The resulting venous hypertension impairs normal venous outflow, leading to chronic venous congestion, spinal cord edema, reduced tissue perfusion, and ultimately ischemic injury. This manifests as progressive myelopathy, termed venous congestive myelopathy (VCM) [2]. In some cases, prolonged or severe venous congestion may result in secondary spinal cord infarction (SCI) [3]. Neurological impairment in SDAVF is primarily due to this congestive mechanism and possible infarction, rather than hemorrhage, which is rare [2].

The clinical onset of SDAVF is typically insidious, with gradually progressive neurological deficits [4]. Common symptoms include lower extremity weakness, sensory disturbances (paresthesia, hypoesthesia), gait difficulties, and back pain [1]. Bowel and bladder dysfunction usually occur later in the disease course [2]. Although slow progression over months or years is characteristic, acute presentations or sudden deterioration, sometimes triggered by physical exertion, specific maneuvers, or secondary infarction, can also occur [5].

A major diagnostic challenge lies in the frequent misdiagnosis of SDAVF [6]. Its nonspecific clinical features often lead to initial consideration of more common conditions, such as degenerative spinal disease, inflammatory transverse myelitis (TM), SCI, chronic inflammatory demyelinating polyneuropathy, or spinal neoplasms. This diagnostic ambiguity is further compounded by the fact that patients often first present to specialties outside neurology or neurosurgery, such as orthopedics, where awareness of SDAVF may be limited [7]. Such diagnostic delays, often extending for months, are associated with poorer neurological outcomes due to irreversible spinal cord injury from chronic venous congestion and possible secondary infarction prior to treatment [8]. The pathophysiology of VCM, marked by gradual onset due to rising venous pressure, contrasts with the abrupt necrosis seen in primary arterial SCI [3]. This distinction makes SDAVF a clinical mimic of subacute inflammatory or chronic degenerative disorders, yet one that may be complicated by infarction.

Magnetic resonance imaging (MRI) is the primary non-invasive diagnostic tool. Characteristic findings of SDAVF include longitudinally extensive T2-weighted hyperintensity within the spinal cord (indicative of edema), often involving the conus medullaris, with cord expansion and dilated, tortuous perimedullary veins seen as serpentine flow voids, most notably along the dorsal cord surface. Parenchymal or venous contrast enhancement may also be present. However, these flow voids can be subtle or absent, adding to diagnostic uncertainty [9]. In cases with secondary infarction, diffusion-weighted imaging (DWI) may show restricted diffusion, a finding usually absent in uncomplicated VCM [3,10]. Magnetic resonance angiography (MRA), especially with time-resolved contrast-enhanced sequences (4D-MRA), improves non-invasive visualization of the fistula and surrounding vasculature, with high sensitivity and specificity [11-13]. Nonetheless, digital subtraction angiography (DSA) remains the gold standard for definitive diagnosis, precise localization of the fistula, and detailed assessment of angioarchitecture essential for treatment planning [1].

Management focuses on obliterating the arteriovenous shunt to reduce venous hypertension and halt disease progression [14]. Treatment options include microsurgical ligation of the draining vein or endovascular embolization of the fistula [15].

## Case presentation

Initial presentation

A 70-year-old man presented to the emergency department with sudden-onset bilateral lower limb weakness, superimposed on a four-week history of bilateral leg spasms, paresthesia ("pins and needles"), and gait unsteadiness. His medical history included poorly controlled hypertension, a 45-pack-year smoking history, and significant regular alcohol consumption. He admitted to inconsistent adherence to prescribed medications, including amlodipine 10 mg daily and atorvastatin 40 mg daily. There was no personal or family history of coagulopathy or illicit drug use.

Upon initial assessment, his blood pressure was elevated at 181/96 mmHg, his heart rate was 86 beats per minute, and his temperature was 36.7°C. Neurological examination revealed marked flaccid weakness in the lower limbs, graded 2/5 on the Medical Research Council (MRC) scale. Deep tendon reflexes were brisk in the lower extremities, and bilateral plantar responses were extensor. A sensory level to pinprick was noted at approximately the T10 level. Urgent bladder ultrasound revealed approximately 1000 mL of retained urine, requiring urinary catheterization. The patient was admitted for further investigation and management.

Investigations

**Figure 1:**
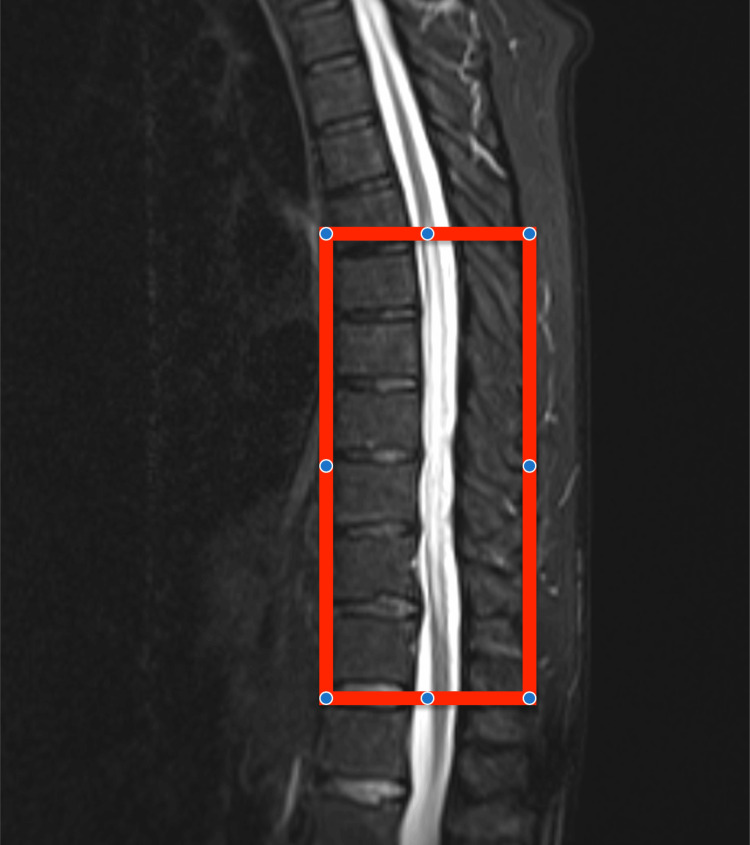
Initial MRI showing a longitudinally extensive T2 hyperintense lesion spanning T7-T11.

Initial investigations focused on identifying the cause of the acute myelopathy. MRI of the whole spine revealed a longitudinally extensive T2 hyperintense lesion extending from T7 to T11, with equivocal gadolinium enhancement. DWI sequences were technically suboptimal. Notably, characteristic MRI features of SDAVF, such as prominent perimedullary flow voids, were not clearly visualized on this initial study, a known factor that can contribute to misdiagnosis [9] (Figure 1).

**Figure 2:**
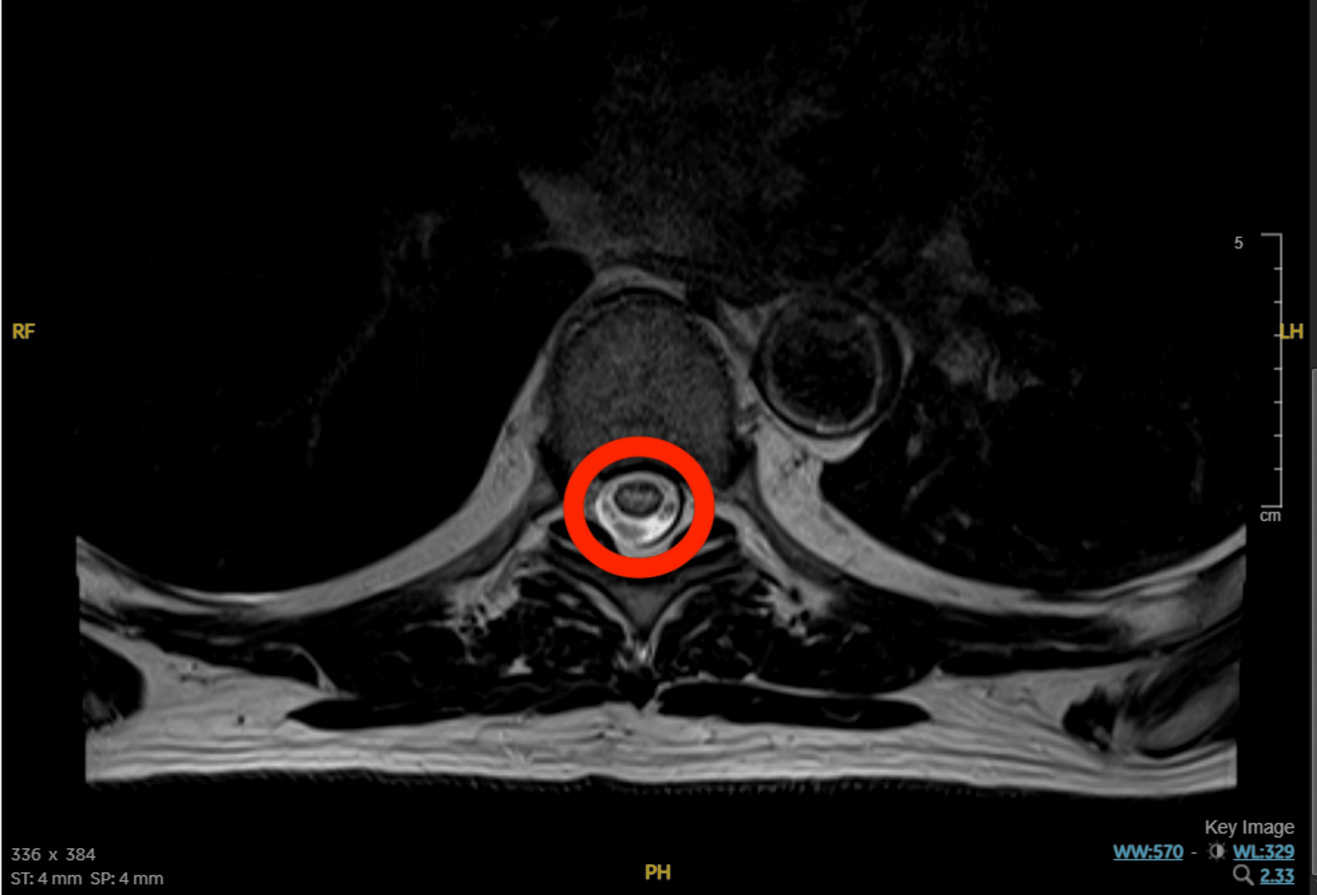
MRI image showing "owl's eye" sign (circled in red).

A repeat MRI was performed due to the lack of clinical improvement. Optimized DWI demonstrated restricted diffusion in the anterior spinal cord from T7 to T11, along with the characteristic “owl’s eye” sign on axial T2-weighted images [3,10] (Figure 2). The “owl’s eye” sign refers to bilateral areas of high signal intensity in the anterior horns of the spinal cord on T2-weighted images and is suggestive of SCI.

Pertinent laboratory investigations, including serum vitamin B12, folate, and homocysteine levels, were within normal limits. Serological screening for syphilis and viral pathogens associated with myelitis, including HIV, yielded negative results. Cerebrospinal fluid (CSF) obtained via lumbar puncture was acellular, with elevated protein (1155 mg/L) and normal glucose concentrations. Oligoclonal bands were absent, and CSF polymerase chain reaction (PCR) assays for relevant viral pathogens were negative. Serological testing for neuromyelitis optica spectrum disorder (NMOSD)-associated aquaporin-4 (AQP4) antibodies and myelin oligodendrocyte glycoprotein (MOG) antibodies also returned negative results [16]. The absence of CSF inflammatory markers argued against typical inflammatory myelitis, but was consistent with findings described in VCM or SCI [2,3].

The combination of acute neurological deterioration superimposed on several weeks of progressive symptoms contributed to diagnostic uncertainty at initial presentation. While abrupt onset typically suggests a vascular etiology such as primary SCI [17], the preceding subacute course is characteristic of VCM secondary to SDAVF, which may present with acute worsening due to secondary infarction [5]. This mixed temporal profile complicated the diagnostic process.

Subsequent presentation

Six months after his initial presentation, the patient returned with marked clinical deterioration. He reported progressive bilateral lower limb weakness over the intervening period. Acutely, he experienced sharp, shooting pains radiating down both legs when attempting to bend forward, which precipitated a fall. His mobility had further declined since hospital discharge.

Due to this clinical worsening, a repeat MRI of the thoracic spine was performed approximately five months after the initial scan. Comparative imaging revealed interval progression of the extensive T2 hyperintensity and cord edema. The spinal cord showed a slight increase in anteroposterior diameter, and the T2 signal abnormality extended from the T6 inferior endplate to the T12 superior endplate. The hyperintensity remained predominantly central. No definite pathological intraspinal enhancement was seen following contrast administration. Importantly, even in this follow-up study, there was no definitive evidence of abnormal serpentine flow voids or engorged intraspinal vessels on T2-weighted or post-contrast sequences to indicate SDAVF.

The progression of cord edema and T2 signal changes over several months was strongly suggestive of an ongoing pathological process. This imaging evolution is highly characteristic of untreated VCM secondary to SDAVF, in which chronic venous hypertension leads to persistent cord edema and progressive ischemic damage, potentially culminating in infarction. The absence of visible flow voids on standard MRI sequences highlights a known limitation of the modality; such findings are not consistently present and may be too subtle to detect without high clinical suspicion or advanced imaging techniques [18].

Given the high suspicion of underlying vascular pathology despite non-specific MRI findings, spinal DSA was performed. This definitive investigation confirmed the presence of a spinal dural arteriovenous fistula originating at the T10-T11 level on the left side, thereby confirming the diagnosis.

Differential diagnosis

The diagnostic pathway in this case evolved significantly, reflecting the inherent challenges in differentiating SDAVF from its clinical mimics.

The initial leading diagnoses included acute TM (ATM) and primary SCI. However, the final confirmed diagnosis was VCM with secondary SCI due to SDAVF. This diagnosis was established by integrating the patient’s clinical trajectory, imaging progression, and definitive angiographic findings [1]. Key supporting factors included the insidious symptom onset followed by acute deterioration, lack of response to corticosteroids [19], progressive MRI changes indicative of chronic venous congestion, and DSA confirmation of a T10-T11 SDAVF. Although the initial DWI was inconclusive, the persistence and severity of neurological deficits following intervention were consistent with irreversible injury due to secondary infarction superimposed on chronic VCM [8]. Taken together, the clinical features, such as progressive multisystem myelopathy, upper motor neuron signs, characteristic MRI evolution (despite the absence of visible flow voids), and the exclusion of alternative etiologies, pointed conclusively to VCM complicated by infarction, secondary to an underlying SDAVF [3,8]. Alternative diagnoses that were considered and subsequently ruled out included compressive myelopathy, demyelinating diseases such as multiple sclerosis and neuromyelitis optica spectrum disorder (NMOSD), and Guillain-Barré syndrome (GBS).

Treatment

Initial management was based on a provisional diagnosis of ATM. The patient received high-dose intravenous methylprednisolone (1 g daily for five days). Due to the absence of clinical improvement, corticosteroid therapy was discontinued. In retrospect, corticosteroid administration in patients with underlying SDAVF may be detrimental; although standard in inflammatory myelitis, steroids can potentially exacerbate venous hypertension and worsen neurological status in SDAVF [19].

Pending diagnostic clarification, subsequent management focused on optimizing vascular risk factors, in line with secondary stroke prevention strategies. Antiplatelet therapy with aspirin (150 mg daily) was initiated, and lipid-lowering therapy was intensified with atorvastatin (80 mg daily) [20,21]. Antihypertensive regimens were adjusted to improve blood pressure control. Counseling was provided regarding alcohol moderation and smoking cessation. Given the history of significant alcohol intake, an alcohol withdrawal protocol was initiated, including intravenous thiamine followed by oral supplementation.

Following angiographic confirmation of the T10-T11 SDAVF, definitive treatment aimed at fistula obliteration was undertaken. The patient first underwent endovascular embolization (coiling), followed by surgical disconnection [15]. While endovascular techniques offer a less invasive approach, surgical interruption generally provides higher rates of complete and durable occlusion with lower recurrence risk [15]. Early and specialized rehabilitation is essential to optimize functional recovery following SDAVF treatment, especially in cases complicated by infarction [8].

Outcome and follow-up

Following successful obliteration of the SDAVF and a course of intensive inpatient rehabilitation, the patient demonstrated partial neurological recovery, particularly in lower limb motor function. However, significant functional impairments persisted, including impaired ambulation requiring walking aids, persistent sensory deficits, and neurogenic bladder dysfunction, all of which adversely impacted his overall quality of life.

This clinical outcome aligns with findings from published literature on SDAVF. Although closure of the fistula halts further progression of VCM by normalizing venous pressure [14], the extent of neurological recovery largely depends on the degree of irreversible ischemic damage sustained prior to treatment [14,22].

The patient’s long-term prognosis remained guarded, reflecting the severity of spinal cord injury. Ongoing care focused on secondary prevention through strict control of vascular risk factors, along with regular outpatient follow-up involving neurology and rehabilitation services.

## Discussion

This case report highlights the diagnostic challenges posed by SDAVF, an uncommon yet treatable etiology of progressive myelopathy [1], which may be further complicated by secondary spinal cord infarction. The patient's clinical course illustrates how VCM resulting from SDAVF, potentially progressing to infarction, can mimic both ATM and primary SCI, contributing to diagnostic delays and adversely affecting long-term outcomes.

The initial presentation combining acute and subacute features, alongside significant vascular risk factors, understandably raised suspicion for SCI or TM. However, the lack of corticosteroid response, non-inflammatory CSF profile, and subsequent clinical and radiological deterioration argued against these initial diagnoses [16,19].

Several factors likely contributed to the diagnostic delay. The mixed temporal onset created ambiguity. Initial MRI, despite demonstrating significant myelopathy, lacked the classic serpentine flow voids frequently associated with SDAVF [9]. Additionally, initial DWI sequences were technically inadequate for evaluating acute arterial SCI [3]. This constellation of non-specific initial findings may have resulted in diagnostic anchoring, delaying consideration of SDAVF until clinical progression compelled reassessment [6,7].

Evolution of the patient's clinical presentation and the progressive MRI abnormalities observed over five months align closely with the pathophysiology of untreated VCM. The T10-T11 SDAVF induced chronic venous hypertension within the peri-medullary plexus, resulting in impaired drainage, cord edema, and progressive ischemic dysfunction, which likely advanced to secondary infarction, primarily impacting the vulnerable lower thoracic cord and conus medullaris [2].

This case reinforces the limitations of standard MRI in SDAVF diagnosis and the importance of advanced imaging. The absence of identifiable flow voids on conventional MRI sequences is noteworthy; their absence does not exclude SDAVF [18]. Other MRI features of restricted diffusion on DWI are characteristic of acute arterial SCI and typically absent in uncomplicated VCM but may be present if secondary infarction occurs [3,10]. Non-invasive contrast-enhanced MRA techniques offer a valuable diagnostic adjunct [11-13]. Nevertheless, DSA remains the indispensable gold standard for confirmation and therapeutic planning [1].

Management strategy evolved congruently with the diagnosis. Empiric steroid therapy proved ineffective and potentially hazardous [19]. Subsequent fistula obliteration via a combined endovascular and surgical strategy addressed the underlying hemodynamic derangement. Current evidence suggests surgical ligation provides superior long-term cure rates compared to endovascular embolization for most SDAVFs [15]. Comprehensive rehabilitation remains critical post-intervention [8].

The potential for iatrogenic neurological worsening following interventions such as high-dose steroids or lumbar puncture in undiagnosed SDAVF necessitates caution [19]. Consideration and exclusion of SDAVF via appropriate imaging should precede potentially harmful interventions in ambiguous myelopathies. Structured diagnostic approaches may facilitate timely diagnosis [23].

Finally, the patient's incomplete recovery highlights the crucial relationship between symptom duration, pretreatment neurological status, and functional outcome, particularly when secondary infarction complicates SDAVF [3,14,22]. Prolonged venous congestion leading to infarction likely caused irreversible neuronal injury. This underscores the need for heightened clinical suspicion to enable early diagnosis and intervention, optimizing the potential for neurological recovery.

## Conclusions

Clear lessons emerged from this case. It underscores the risk of misdiagnosis when differentiating myelopathies based solely on initial clinical presentation or standard imaging. Importantly, it highlights the need for a high index of suspicion for SDAVF in patients with progressive, atypical, or treatment-refractory myelopathy. In this case, longitudinal clinical monitoring, reassessment of treatment response, and recognition of evolving MRI changes were crucial in shifting the diagnostic focus from TM and primary SCI to VCM complicated by secondary infarction, ultimately leading to the definitive diagnosis via spinal DSA.
